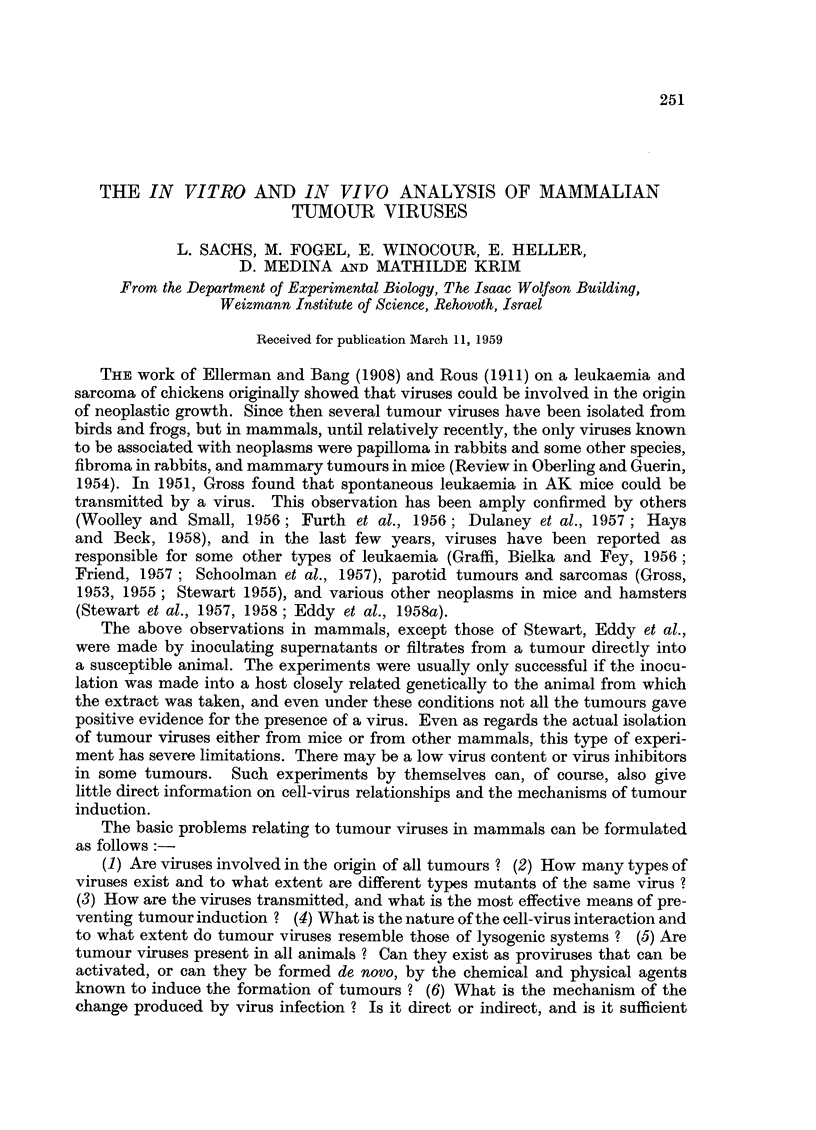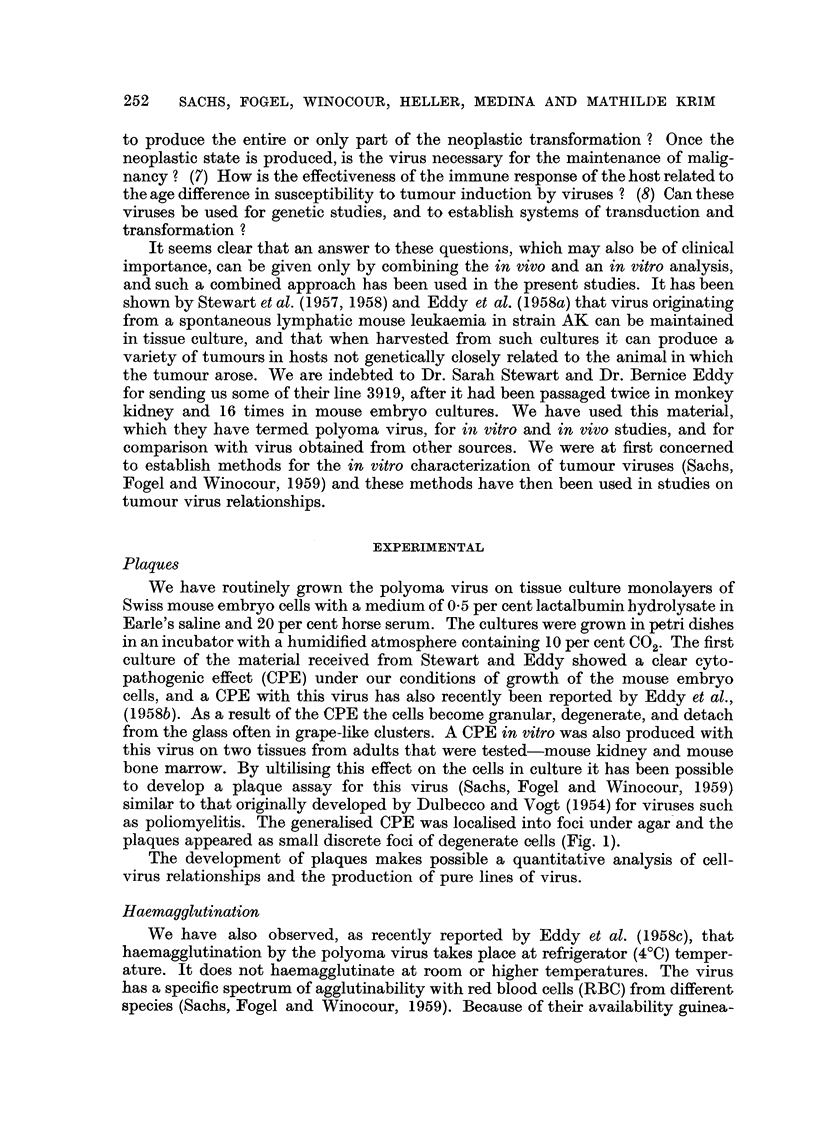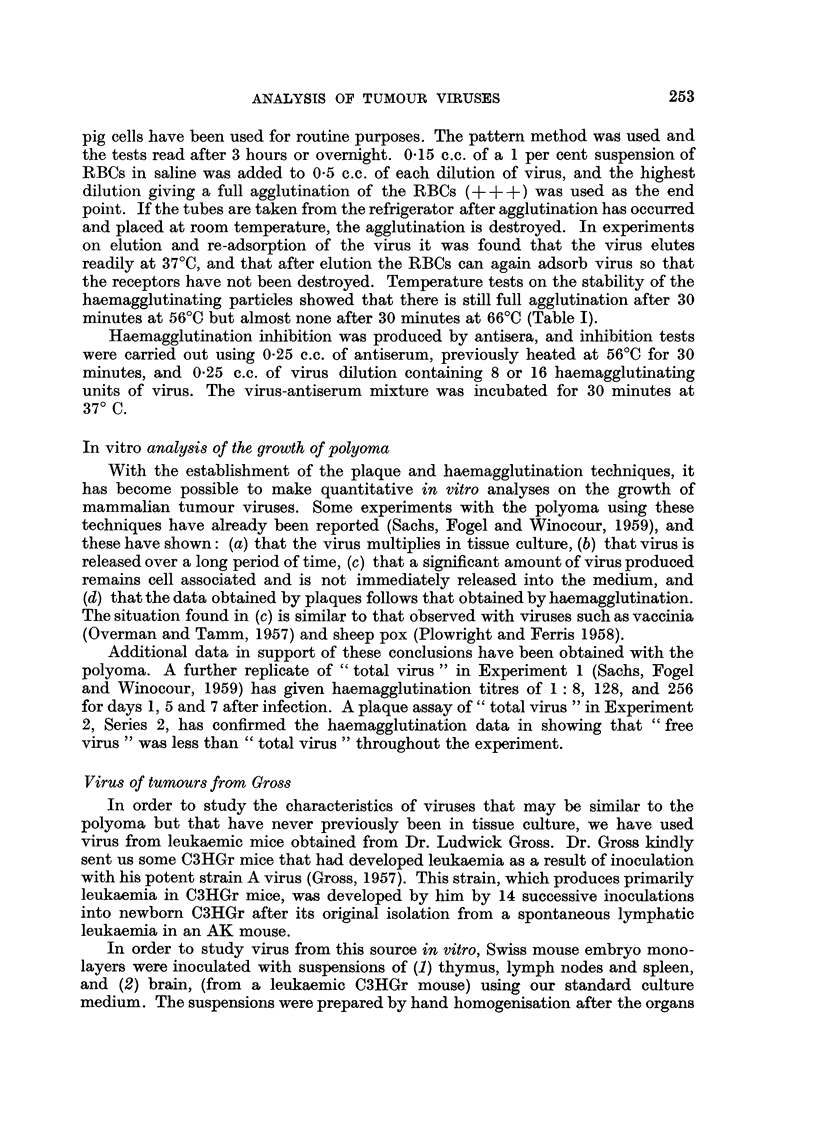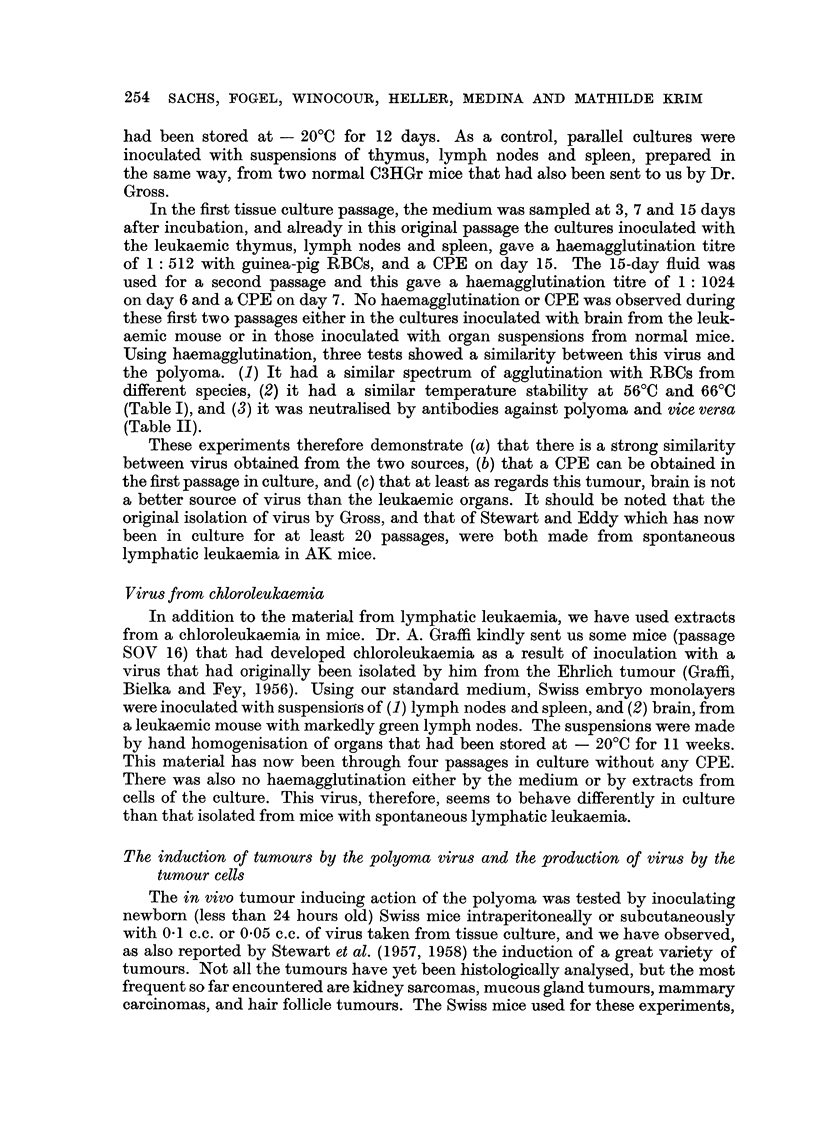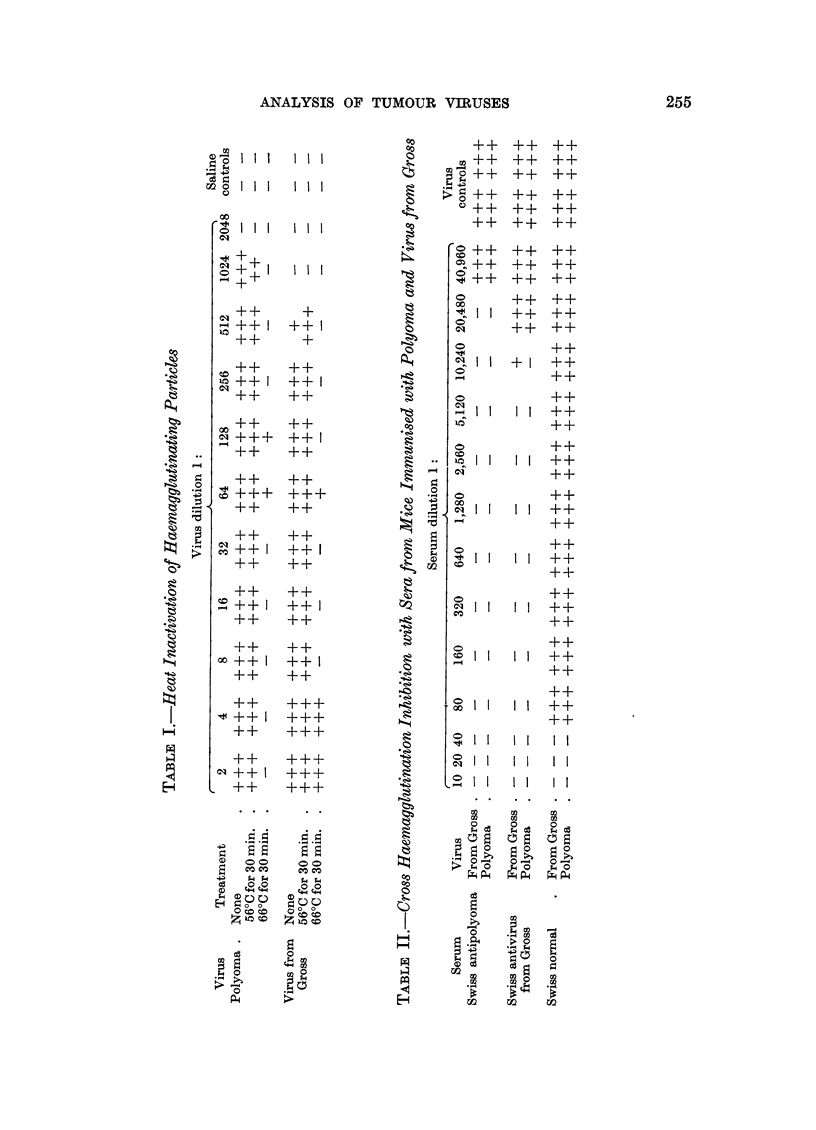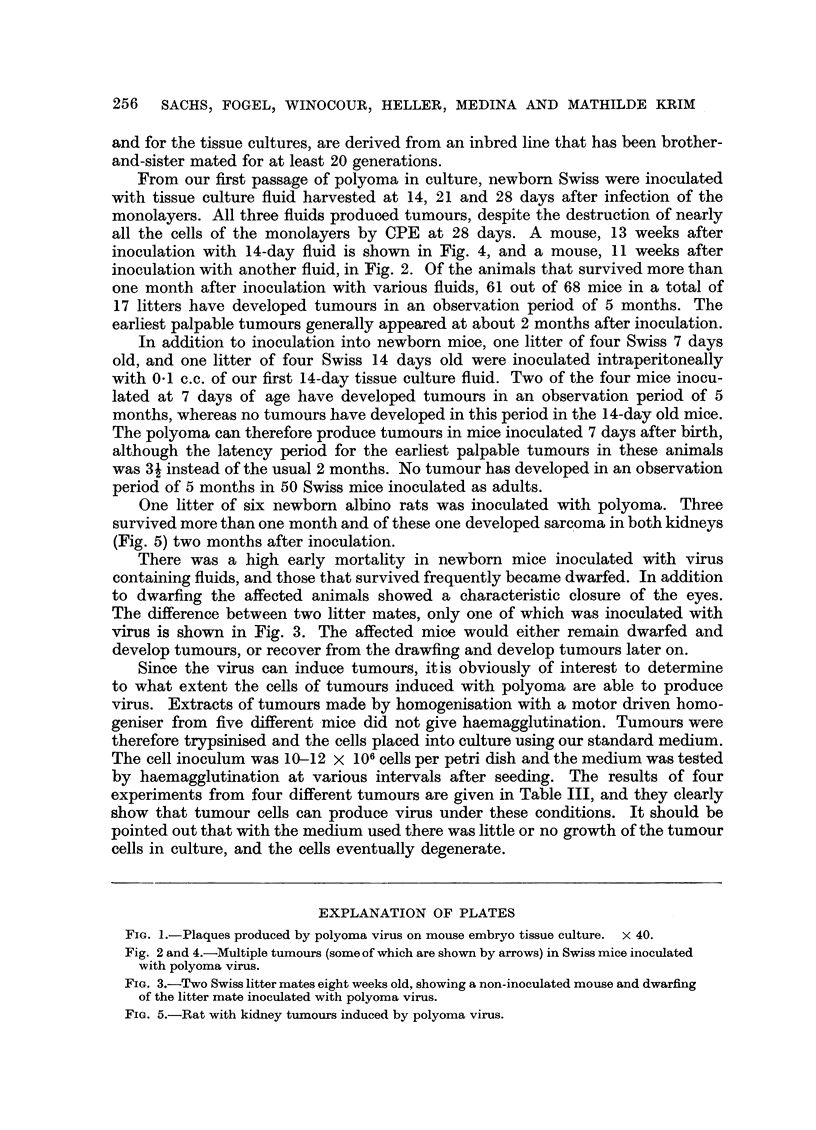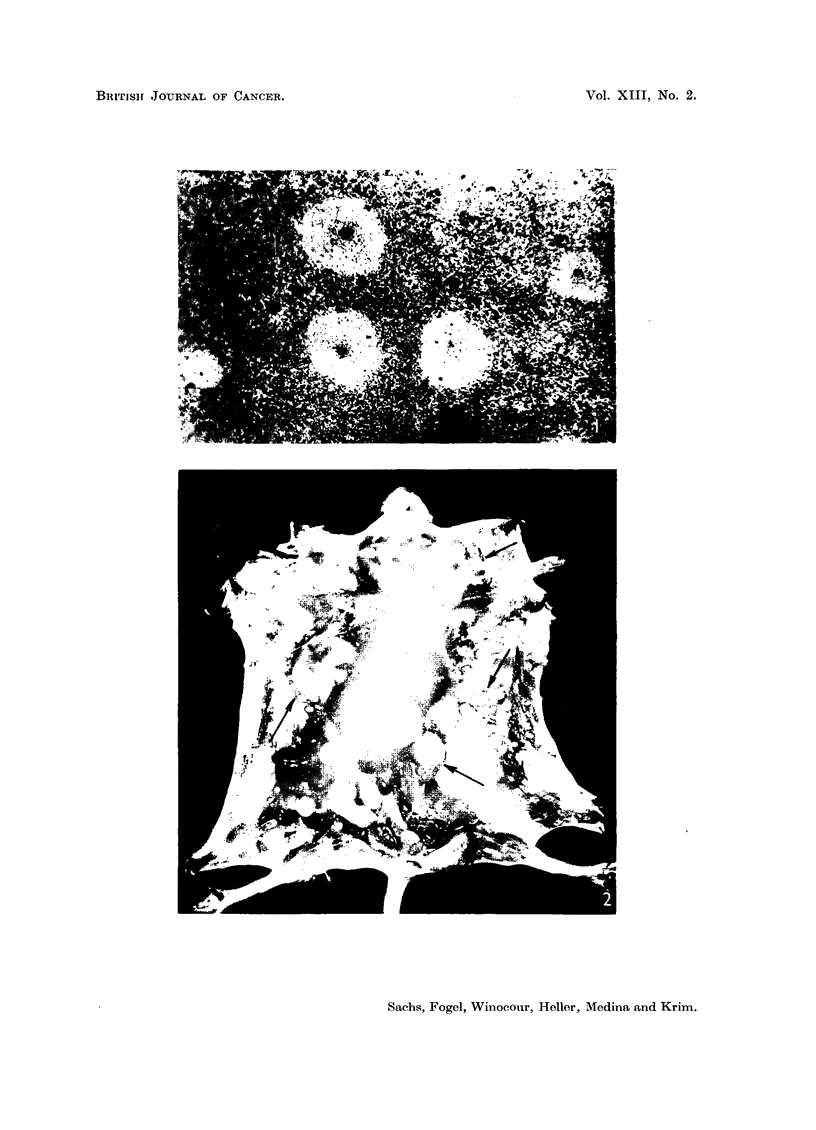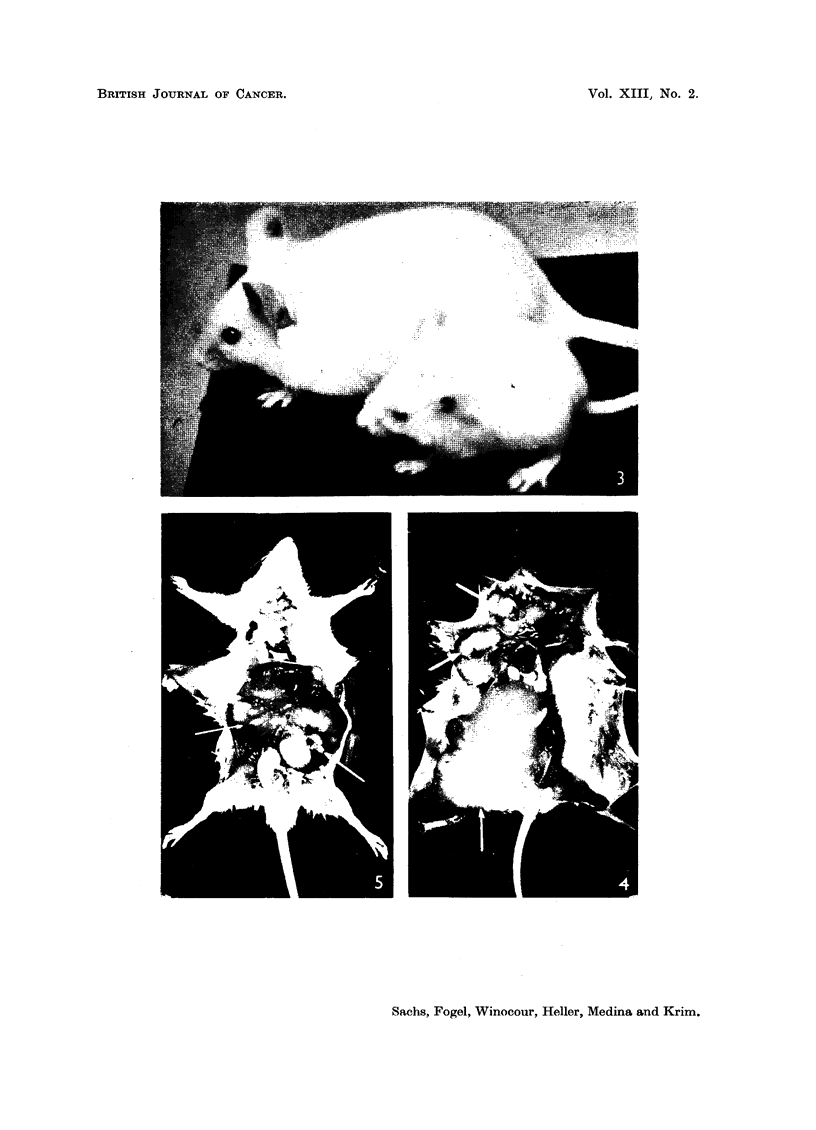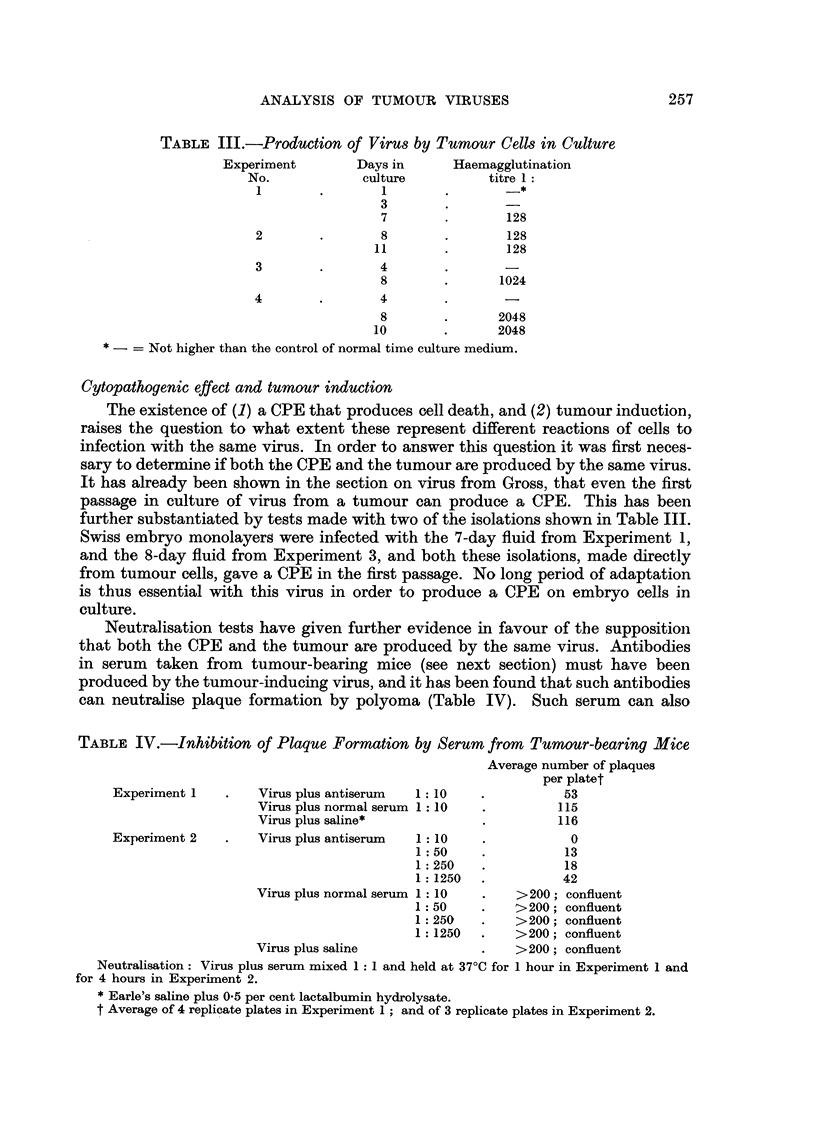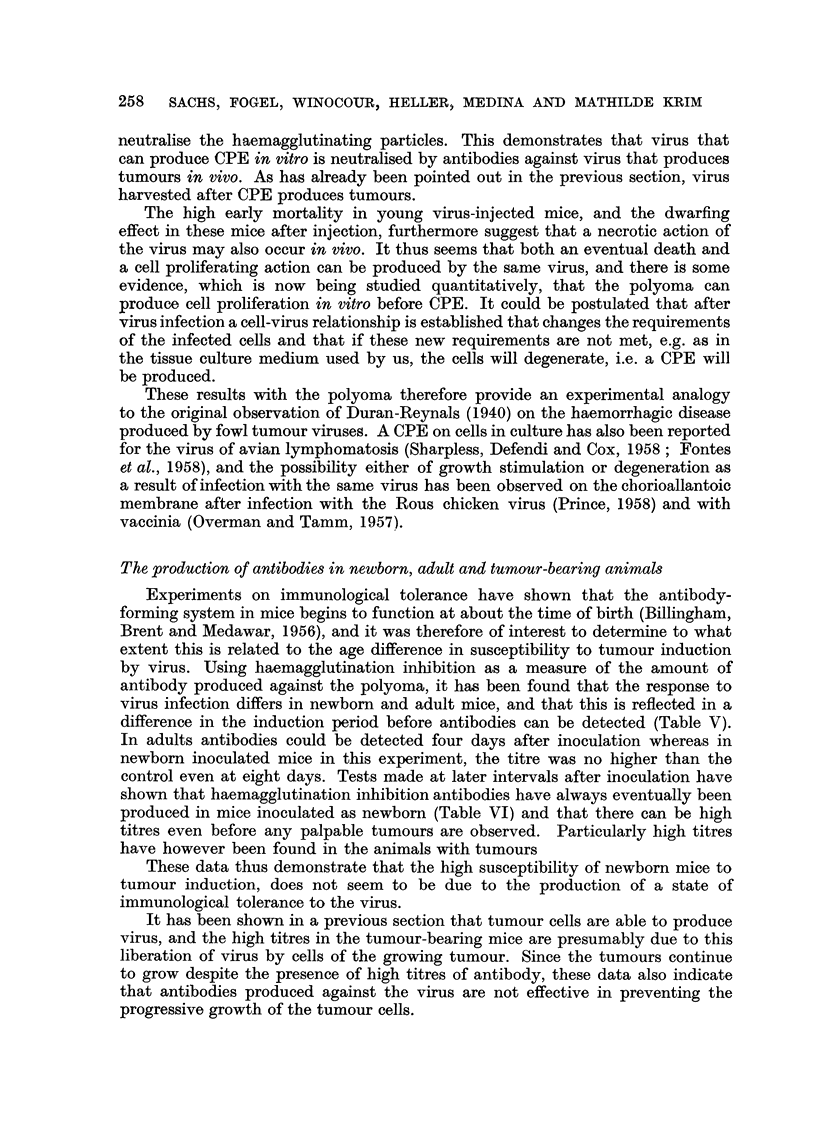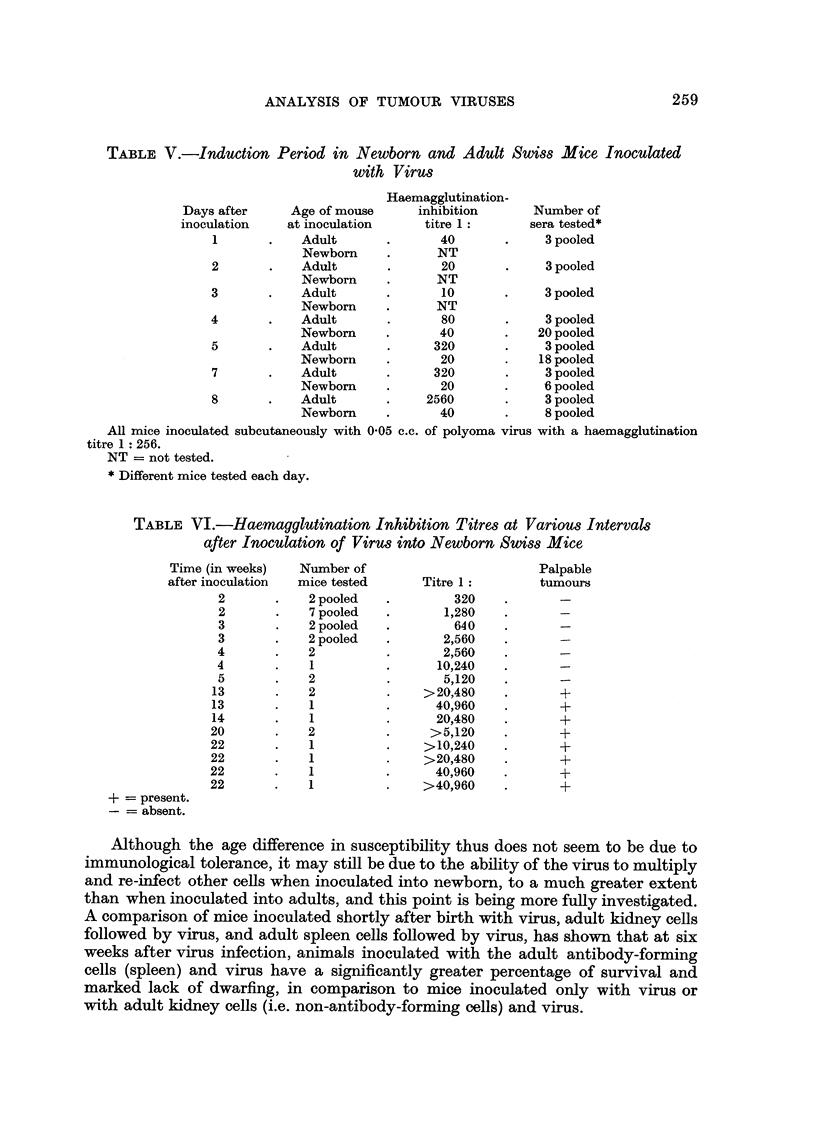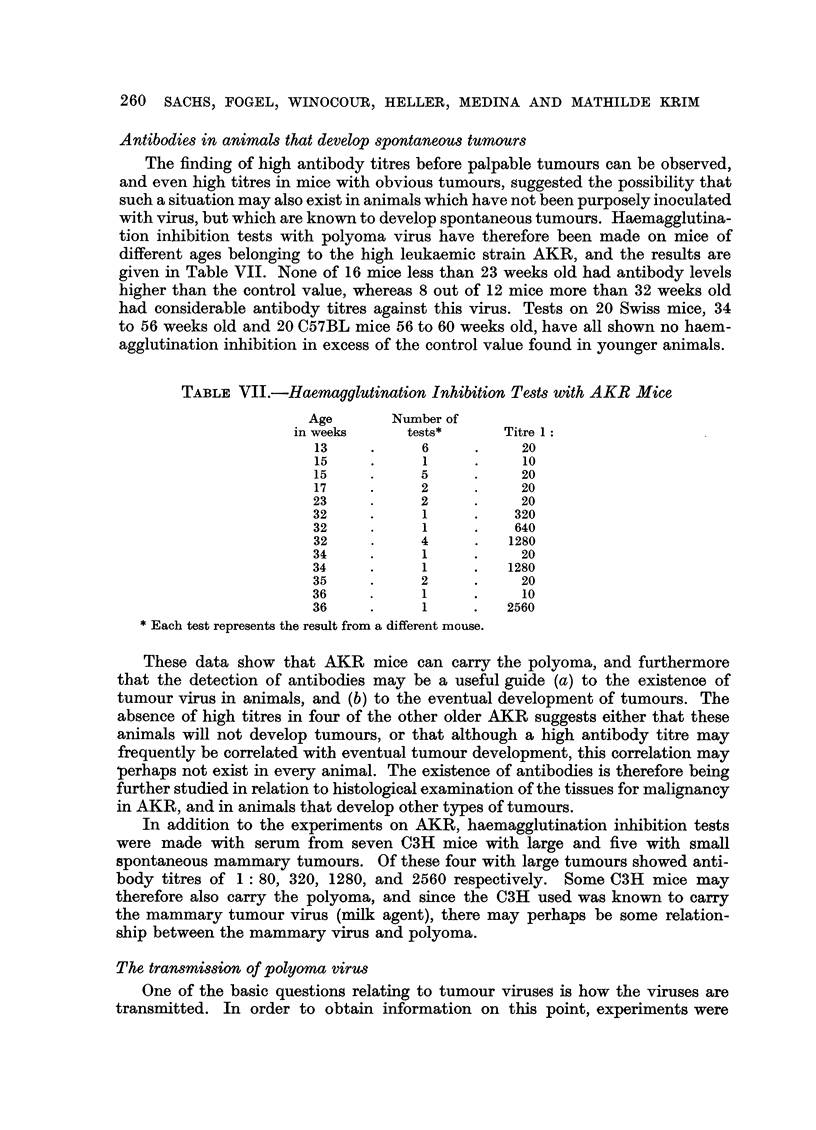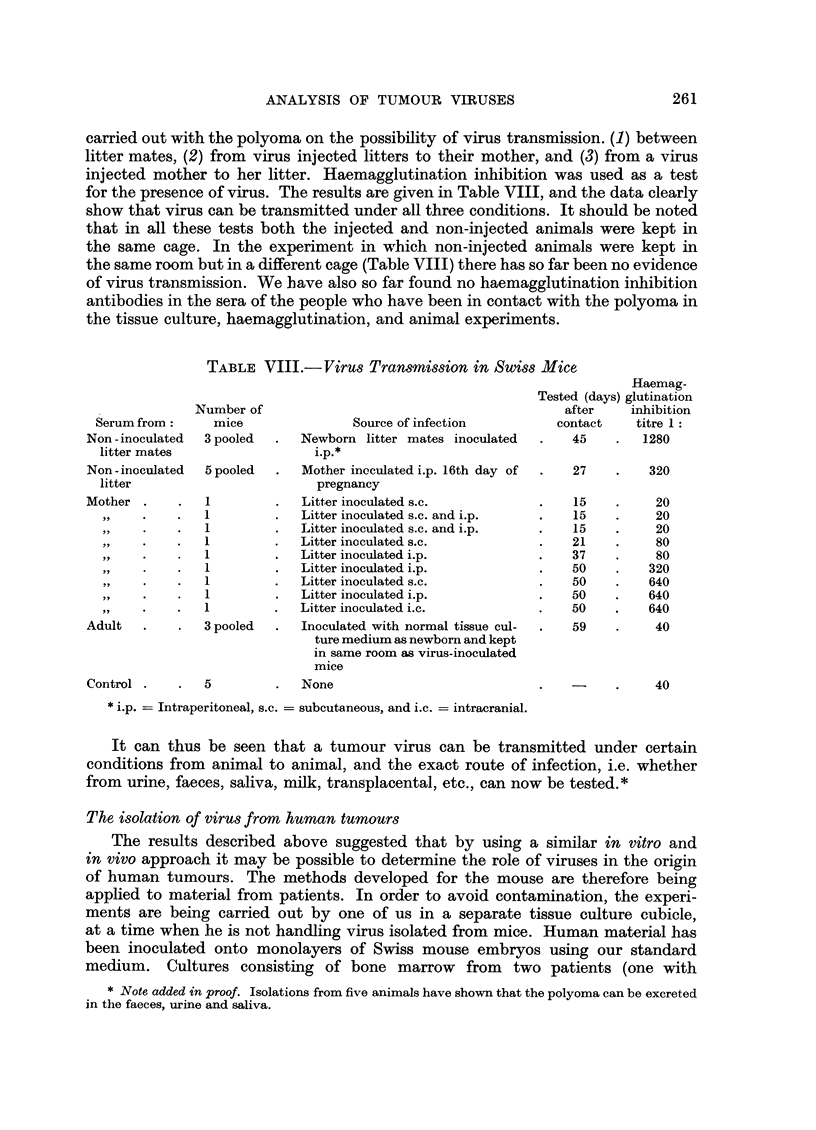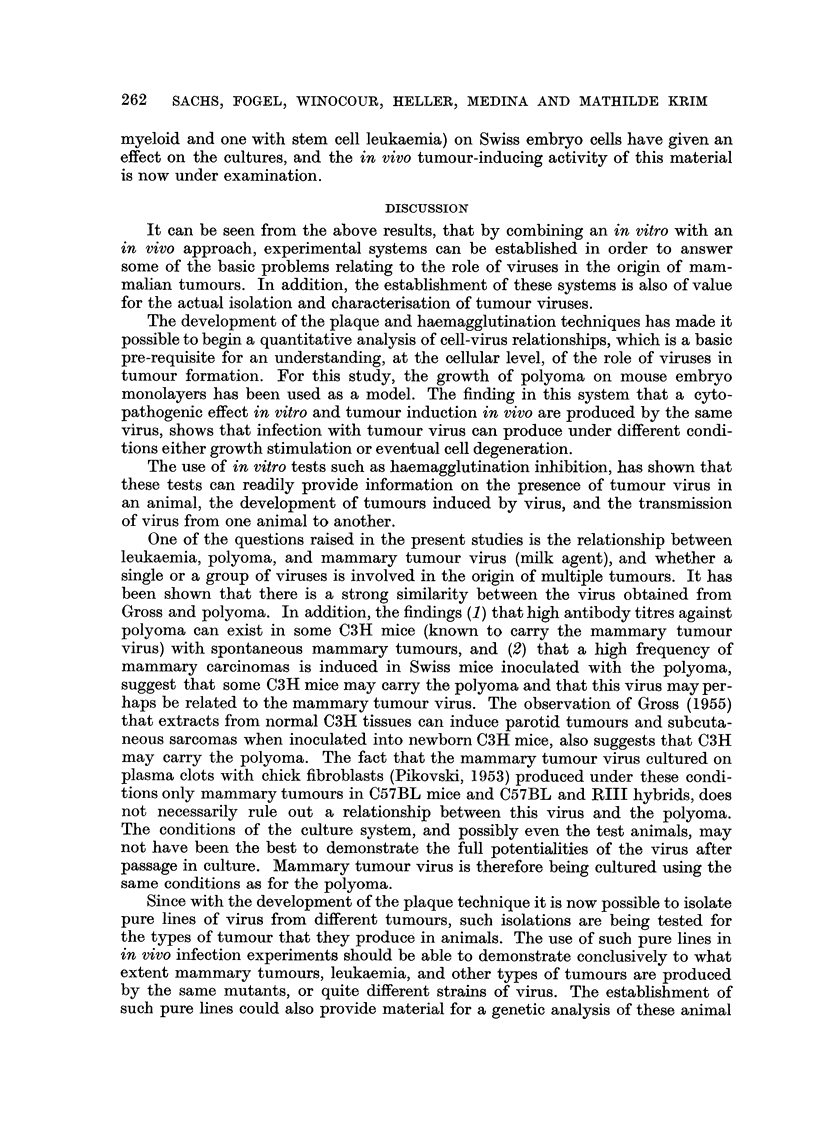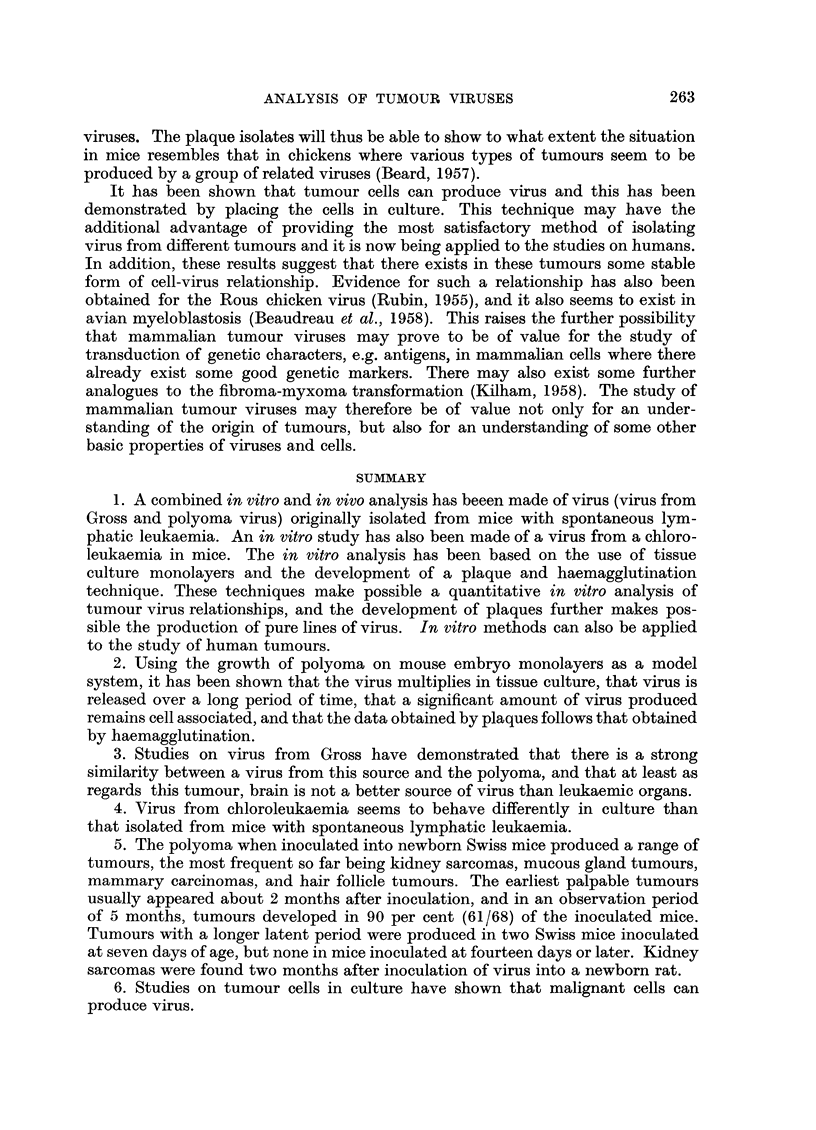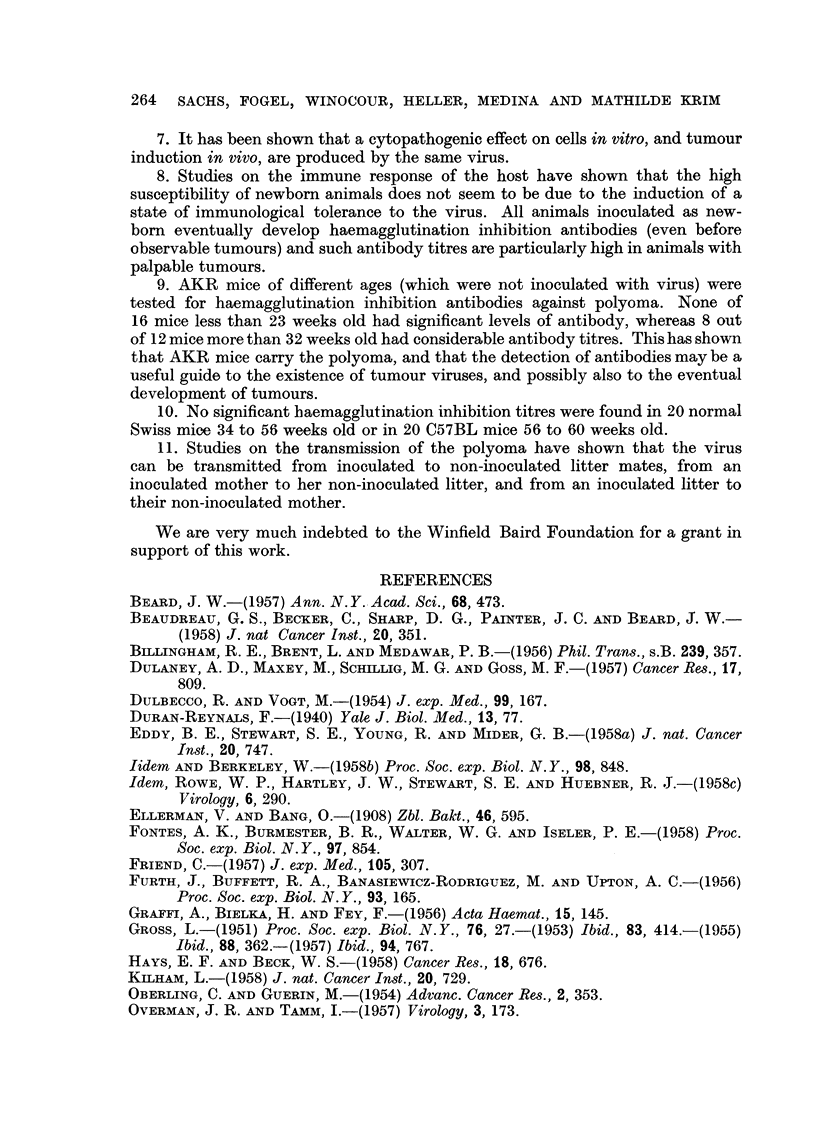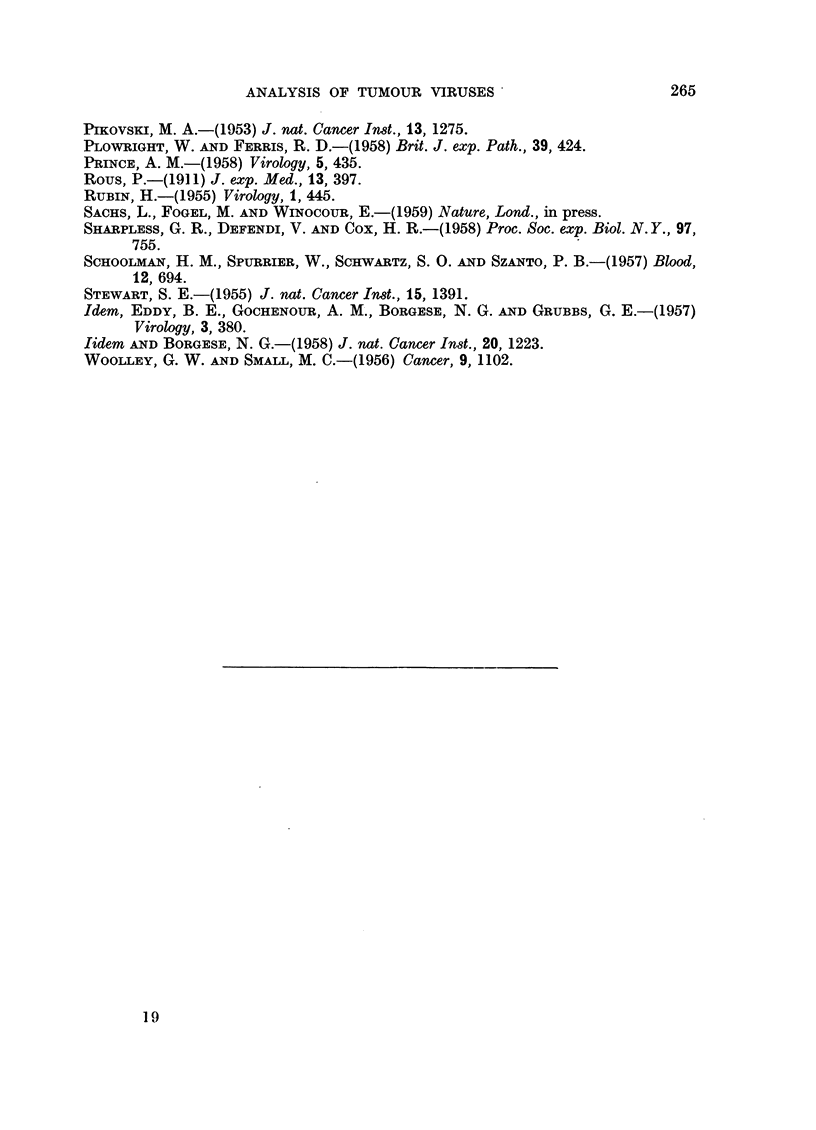# The In Vitro and In Vivo Analysis of Mammalian Tumour Viruses

**DOI:** 10.1038/bjc.1959.32

**Published:** 1959-06

**Authors:** L. Sachs, M. Fogel, E. Winocour, E. Heller, D. Medina, Mathilde Krim

## Abstract

**Images:**


### THE *IN VITRO* AND *IN VIVO* ANALYSIS OF MAMMALIAN TUMOUR VIRUSES

L. SACHS, M. FOGEL, E. WINOCOUR, E. HELLER, D. MEDINA AND MATHILDE KRIM

*From the Department of Experimental Biology, The Isaac Wolfson Building, Weizmann Institute of Science, Rehovoth, Israel*

Received for publication March 11, 1959

THE work of Ellerman and Bang (1908) and Rous (1911) on a leukaemia and sarcoma of chickens originally showed that viruses could be involved in the origin of neoplastic growth. Since then several tumour viruses have been isolated from birds and frogs, but in mammals, until relatively recently, the only viruses known to be associated with neoplasms were papilloma in rabbits and some other species, fibroma in rabbits, and mammary tumours in mice (Review in Oberling and Guerin, 1954). In 1951, Gross found that spontaneous leukaemia in AK mice could be transmitted by a virus. This observation has been amply confirmed by others (Woolley and Small, 1956; Furth *et al.*, 1956; Dulaney *et al.*, 1957; Hays and Beck, 1958), and in the last few years, viruses have been reported as responsible for some other types of leukaemia (Graffi, Bielka and Fey, 1956; Friend, 1957; Schoolman *et al.*, 1957), parotid tumours and sarcomas (Gross, 1953, 1955; Stewart 1955), and various other neoplasms in mice and hamsters (Stewart *et al.*, 1957, 1958; Eddy *et al.*, 1958*a*).

The above observations in mammals, except those of Stewart, Eddy *et al.*, were made by inoculating supernatants or filtrates from a tumour directly into a susceptible animal. The experiments were usually only successful if the inoculation was made into a host closely related genetically to the animal from which the extract was taken, and even under these conditions not all the tumours gave positive evidence for the presence of a virus. Even as regards the actual isolation of tumour viruses either from mice or from other mammals, this type of experiment has severe limitations. There may be a low virus content or virus inhibitors in some tumours. Such experiments by themselves can, of course, also give little direct information on cell-virus relationships and the mechanisms of tumour induction.

The basic problems relating to tumour viruses in mammals can be formulated as follows :—

(*1*) Are viruses involved in the origin of all tumours ? (*2*) How many types of viruses exist and to what extent are different types mutants of the same virus ? (*3*) How are the viruses transmitted, and what is the most effective means of preventing tumour induction ? (*4*) What is the nature of the cell-virus interaction and to what extent do tumour viruses resemble those of lysogenic systems ? (*5*) Are tumour viruses present in all animals ? Can they exist as proviruses that can be activated, or can they be formed *de novo*, by the chemical and physical agents known to induce the formation of tumours ? (*6*) What is the mechanism of the change produced by virus infection ? Is it direct or indirect, and is it sufficient to produce the entire or only part of the neoplastic transformation ? Once the neoplastic state is produced, is the virus necessary for the maintenance of malignancy ? (*7*) How is the effectiveness of the immune response of the host related to the age difference in susceptibility to tumour induction by viruses ? (*8*) Can these viruses be used for genetic studies, and to establish systems of transduction and transformation ?

It seems clear that an answer to these questions, which may also be of clinical importance, can be given only by combining the *in vivo* and an *in vitro* analysis, and such a combined approach has been used in the present studies. It has been shown by Stewart *et al.* (1957, 1958) and Eddy *et al.* (1958*a*) that virus originating from a spontaneous lymphatic mouse leukaemia in strain AK can be maintained in tissue culture, and that when harvested from such cultures it can produce a variety of tumours in hosts not genetically closely related to the animal in which the tumour arose. We are indebted to Dr. Sarah Stewart and Dr. Bernice Eddy for sending us some of their line 3919, after it had been passaged twice in monkey kidney and 16 times in mouse embryo cultures. We have used this material, which they have termed polyoma virus, for *in vitro* and *in vivo* studies, and for comparison with virus obtained from other sources. We were at first concerned to establish methods for the *in vitro* characterization of tumour viruses (Sachs, Fogel and Winocour, 1959) and these methods have then been used in studies on tumour virus relationships.

### EXPERIMENTAL

### *Plaques*

We have routinely grown the polyoma virus on tissue culture monolayers of Swiss mouse embryo cells with a medium of 0·5 per cent lactalbumin hydrolysate in Earle's saline and 20 per cent horse serum. The cultures were grown in petri dishes in an incubator with a humidified atmosphere containing 10 per cent $CO_2$. The first culture of the material received from Stewart and Eddy showed a clear cytopathogenic effect (CPE) under our conditions of growth of the mouse embryo cells, and a CPE with this virus has also recently been reported by Eddy *et al.*, (1958*b*). As a result of the CPE the cells become granular, degenerate, and detach from the glass often in grape-like clusters. A CPE *in vitro* was also produced with this virus on two tissues from adults that were tested—mouse kidney and mouse bone marrow. By ultilising this effect on the cells in culture it has been possible to develop a plaque assay for this virus (Sachs, Fogel and Winocour, 1959) similar to that originally developed by Dulbecco and Vogt (1954) for viruses such as poliomyelitis. The generalised CPE was localised into foci under agar and the plaques appeared as small discrete foci of degenerate cells (Fig. 1).

The development of plaques makes possible a quantitative analysis of cell-virus relationships and the production of pure lines of virus.

### *Haemagglutination*

We have also observed, as recently reported by Eddy *et al.* (1958*c*), that haemagglutination by the polyoma virus takes place at refrigerator (4°C) temperature. It does not haemagglutinate at room or higher temperatures. The virus has a specific spectrum of agglutinability with red blood cells (RBC) from different species (Sachs, Fogel and Winocour, 1959). Because of their availability guineapig cells have been used for routine purposes. The pattern method was used and the tests read after 3 hours or overnight. 0·15 c.c. of a 1 per cent suspension of RBCs in saline was added to 0·5 c.c. of each dilution of virus, and the highest dilution giving a full agglutination of the RBCs (+++) was used as the end point. If the tubes are taken from the refrigerator after agglutination has occurred and placed at room temperature, the agglutination is destroyed. In experiments on elution and re-adsorption of the virus it was found that the virus elutes readily at 37°C, and that after elution the RBCs can again adsorb virus so that the receptors have not been destroyed. Temperature tests on the stability of the haemagglutinating particles showed that there is still full agglutination after 30 minutes at 56°C but almost none after 30 minutes at 66°C (Table I).

Haemagglutination inhibition was produced by antisera, and inhibition tests were carried out using 0·25 c.c. of antiserum, previously heated at 56°C for 30 minutes, and 0·25 c.c. of virus dilution containing 8 or 16 haemagglutinating units of virus. The virus-antiserum mixture was incubated for 30 minutes at 37° C.

In vitro *analysis of the growth of polyoma*

With the establishment of the plaque and haemagglutination techniques, it has become possible to make quantitative *in vitro* analyses on the growth of mammalian tumour viruses. Some experiments with the polyoma using these techniques have already been reported (Sachs, Fogel and Winocour, 1959), and these have shown: (*a*) that the virus multiplies in tissue culture, (*b*) that virus is released over a long period of time, (*c*) that a significant amount of virus produced remains cell associated and is not immediately released into the medium, and (*d*) that the data obtained by plaques follows that obtained by haemagglutination. The situation found in (*c*) is similar to that observed with viruses such as vaccinia (Overman and Tamm, 1957) and sheep pox (Plowright and Ferris 1958).

Additional data in support of these conclusions have been obtained with the polyoma. A further replicate of " total virus " in Experiment 1 (Sachs, Fogel and Winocour, 1959) has given haemagglutination titres of 1 : 8, 128, and 256 for days 1, 5 and 7 after infection. A plaque assay of " total virus " in Experiment 2, Series 2, has confirmed the haemagglutination data in showing that " free virus " was less than " total virus " throughout the experiment.

*Virus of tumours from Gross*

In order to study the characteristics of viruses that may be similar to the polyoma but that have never previously been in tissue culture, we have used virus from leukaemic mice obtained from Dr. Ludwick Gross. Dr. Gross kindly sent us some C3HGr mice that had developed leukaemia as a result of inoculation with his potent strain A virus (Gross, 1957). This strain, which produces primarily leukaemia in C3HGr mice, was developed by him by 14 successive inoculations into newborn C3HGr after its original isolation from a spontaneous lymphatic leukaemia in an AK mouse.

In order to study virus from this source *in vitro*, Swiss mouse embryo monolayers were inoculated with suspensions of (*1*) thymus, lymph nodes and spleen, and (*2*) brain, (from a leukaemic C3HGr mouse) using our standard culture medium. The suspensions were prepared by hand homogenisation after the organs had been stored at − 20°C for 12 days. As a control, parallel cultures were inoculated with suspensions of thymus, lymph nodes and spleen, prepared in the same way, from two normal C3HGr mice that had also been sent to us by Dr. Gross.

In the first tissue culture passage, the medium was sampled at 3, 7 and 15 days after incubation, and already in this original passage the cultures inoculated with the leukaemic thymus, lymph nodes and spleen, gave a haemagglutination titre of 1 : 512 with guinea-pig RBCs, and a CPE on day 15. The 15-day fluid was used for a second passage and this gave a haemagglutination titre of 1 : 1024 on day 6 and a CPE on day 7. No haemagglutination or CPE was observed during these first two passages either in the cultures inoculated with brain from the leukaemic mouse or in those inoculated with organ suspensions from normal mice. Using haemagglutination, three tests showed a similarity between this virus and the polyoma. (*1*) It had a similar spectrum of agglutination with RBCs from different species, (*2*) it had a similar temperature stability at 56°C and 66°C (Table I), and (*3*) it was neutralised by antibodies against polyoma and *vice versa* (Table II).

These experiments therefore demonstrate (*a*) that there is a strong similarity between virus obtained from the two sources, (*b*) that a CPE can be obtained in the first passage in culture, and (*c*) that at least as regards this tumour, brain is not a better source of virus than the leukaemic organs. It should be noted that the original isolation of virus by Gross, and that of Stewart and Eddy which has now been in culture for at least 20 passages, were both made from spontaneous lymphatic leukaemia in AK mice.

*Virus from chloroleukaemia*

In addition to the material from lymphatic leukaemia, we have used extracts from a chloroleukaemia in mice. Dr. A. Graffi kindly sent us some mice (passage SOV 16) that had developed chloroleukaemia as a result of inoculation with a virus that had originally been isolated by him from the Ehrlich tumour (Graffi, Bielka and Fey, 1956). Using our standard medium, Swiss embryo monolayers were inoculated with suspensions of (*1*) lymph nodes and spleen, and (*2*) brain, from a leukaemic mouse with markedly green lymph nodes. The suspensions were made by hand homogenisation of organs that had been stored at − 20°C for 11 weeks. This material has now been through four passages in culture without any CPE. There was also no haemagglutination either by the medium or by extracts from cells of the culture. This virus, therefore, seems to behave differently in culture than that isolated from mice with spontaneous lymphatic leukaemia.

*The induction of tumours by the polyoma virus and the production of virus by the tumour cells*

The *in vivo* tumour inducing action of the polyoma was tested by inoculating newborn (less than 24 hours old) Swiss mice intraperitoneally or subcutaneously with 0·1 c.c. or 0·05 c.c. of virus taken from tissue culture, and we have observed, as also reported by Stewart *et al.* (1957, 1958) the induction of a great variety of tumours. Not all the tumours have yet been histologically analysed, but the most frequent so far encountered are kidney sarcomas, mucous gland tumours, mammary carcinomas, and hair follicle tumours. The Swiss mice used for these experiments,

TABLE I.—*Heat Inactivation of Haemagglutinating Particles*

| Virus | Treatment | Virus dilution 1 : 2 | 4 | 8 | 16 | 32 | 64 | 128 | 256 | 512 | 1024 | 2048 | Saline controls | |
|---|---|---|---|---|---|---|---|---|---|---|---|---|---|---|
| Polyoma | None | +++ | +++ | +++ | +++ | +++ | +++ | +++ | +++ | +++ | +++ | — | — | — |
| | 56°C for 30 min. | +++ | +++ | +++ | +++ | +++ | +++ | +++ | +++ | +++ | ++ | — | — | — |
| | 66°C for 30 min. | — | — | — | — | — | + | + | — | — | — | — | — | — |
| Virus from Gross | None | +++ | +++ | +++ | +++ | +++ | +++ | +++ | +++ | + | — | — | — | — |
| | 56°C for 30 min. | +++ | +++ | +++ | +++ | +++ | +++ | +++ | +++ | +++ | — | — | — | — |
| | 66°C for 30 min. | +++ | +++ | — | — | — | + | — | — | — | — | — | — | — |

TABLE II.—*Cross Haemagglutination Inhibition with Sera from Mice Immunised with Polyoma and Virus from Gross*

| Serum | Virus | Serum dilution 1 : 10 | 20 | 40 | 80 | 160 | 320 | 640 | 1,280 | 2,560 | 5,120 | 10,240 | 20,480 | 40,960 | Virus controls | |
|---|---|---|---|---|---|---|---|---|---|---|---|---|---|---|---|---|
| Swiss antipolyoma | From Gross | — | — | — | — | — | — | — | — | — | — | — | — | +++ | +++ | +++ |
| | Polyoma | — | — | — | — | — | — | — | — | — | — | — | — | +++ | +++ | +++ |
| Swiss antivirus from Gross | From Gross | — | — | — | — | — | — | — | — | — | — | + | +++ | +++ | +++ | +++ |
| | Polyoma | — | — | — | — | — | — | — | — | — | — | — | +++ | +++ | +++ | +++ |
| Swiss normal | From Gross | — | — | — | +++ | +++ | +++ | +++ | +++ | +++ | +++ | +++ | +++ | +++ | +++ | +++ |
| | Polyoma | — | — | — | +++ | +++ | +++ | +++ | +++ | +++ | +++ | +++ | +++ | +++ | +++ | +++ |

and for the tissue cultures, are derived from an inbred line that has been brother-and-sister mated for at least 20 generations.

From our first passage of polyoma in culture, newborn Swiss were inoculated with tissue culture fluid harvested at 14, 21 and 28 days after infection of the monolayers. All three fluids produced tumours, despite the destruction of nearly all the cells of the monolayers by CPE at 28 days. A mouse, 13 weeks after inoculation with 14-day fluid is shown in Fig. 4, and a mouse, 11 weeks after inoculation with another fluid, in Fig. 2. Of the animals that survived more than one month after inoculation with various fluids, 61 out of 68 mice in a total of 17 litters have developed tumours in an observation period of 5 months. The earliest palpable tumours generally appeared at about 2 months after inoculation.

In addition to inoculation into newborn mice, one litter of four Swiss 7 days old, and one litter of four Swiss 14 days old were inoculated intraperitoneally with 0·1 c.c. of our first 14-day tissue culture fluid. Two of the four mice inoculated at 7 days of age have developed tumours in an observation period of 5 months, whereas no tumours have developed in this period in the 14-day old mice. The polyoma can therefore produce tumours in mice inoculated 7 days after birth, although the latency period for the earliest palpable tumours in these animals was 3½ instead of the usual 2 months. No tumour has developed in an observation period of 5 months in 50 Swiss mice inoculated as adults.

One litter of six newborn albino rats was inoculated with polyoma. Three survived more than one month and of these one developed sarcoma in both kidneys (Fig. 5) two months after inoculation.

There was a high early mortality in newborn mice inoculated with virus containing fluids, and those that survived frequently became dwarfed. In addition to dwarfing the affected animals showed a characteristic closure of the eyes. The difference between two litter mates, only one of which was inoculated with virus is shown in Fig. 3. The affected mice would either remain dwarfed and develop tumours, or recover from the drawfing and develop tumours later on.

Since the virus can induce tumours, itis obviously of interest to determine to what extent the cells of tumours induced with polyoma are able to produce virus. Extracts of tumours made by homogenisation with a motor driven homogeniser from five different mice did not give haemagglutination. Tumours were therefore trypsinised and the cells placed into culture using our standard medium. The cell inoculum was 10–12 $\times$ $10^6$ cells per petri dish and the medium was tested by haemagglutination at various intervals after seeding. The results of four experiments from four different tumours are given in Table III, and they clearly show that tumour cells can produce virus under these conditions. It should be pointed out that with the medium used there was little or no growth of the tumour cells in culture, and the cells eventually degenerate.

---

EXPLANATION OF PLATES

FIG. 1.—Plaques produced by polyoma virus on mouse embryo tissue culture. $\times$ 40.

Fig. 2 and 4.—Multiple tumours (some of which are shown by arrows) in Swiss mice inoculated with polyoma virus.

FIG. 3.—Two Swiss litter mates eight weeks old, showing a non-inoculated mouse and dwarfing of the litter mate inoculated with polyoma virus.

FIG. 5.—Rat with kidney tumours induced by polyoma virus.

Sachs, Fogel, Winocour, Heller, Medina and Krim.

3

5

4

TABLE III.—*Production of Virus by Tumour Cells in Culture*

| Experiment No. | Days in culture | Haemagglutination titre 1 : |
|---|---|---|
| 1 | 1 | —* |
| | 3 | — |
| | 7 | 128 |
| 2 | 8 | 128 |
| | 11 | 128 |
| 3 | 4 | — |
| | 8 | 1024 |
| 4 | 4 | — |
| | 8 | 2048 |
| | 10 | 2048 |

* — = Not higher than the control of normal time culture medium.

*Cytopathogenic effect and tumour induction*

The existence of (*1*) a CPE that produces cell death, and (*2*) tumour induction, raises the question to what extent these represent different reactions of cells to infection with the same virus. In order to answer this question it was first necessary to determine if both the CPE and the tumour are produced by the same virus. It has already been shown in the section on virus from Gross, that even the first passage in culture of virus from a tumour can produce a CPE. This has been further substantiated by tests made with two of the isolations shown in Table III. Swiss embryo monolayers were infected with the 7-day fluid from Experiment 1, and the 8-day fluid from Experiment 3, and both these isolations, made directly from tumour cells, gave a CPE in the first passage. No long period of adaptation is thus essential with this virus in order to produce a CPE on embryo cells in culture.

Neutralisation tests have given further evidence in favour of the supposition that both the CPE and the tumour are produced by the same virus. Antibodies in serum taken from tumour-bearing mice (see next section) must have been produced by the tumour-inducing virus, and it has been found that such antibodies can neutralise plaque formation by polyoma (Table IV). Such serum can also

TABLE IV.—*Inhibition of Plaque Formation by Serum from Tumour-bearing Mice*

| | | | Average number of plaques per plate† |
|---|---|---|---|
| Experiment 1 | Virus plus antiserum | 1 : 10 | 53 |
| | Virus plus normal serum | 1 : 10 | 115 |
| | Virus plus saline* | | 116 |
| Experiment 2 | Virus plus antiserum | 1 : 10 | 0 |
| | | 1 : 50 | 13 |
| | | 1 : 250 | 18 |
| | | 1 : 1250 | 42 |
| | Virus plus normal serum | 1 : 10 | >200 ; confluent |
| | | 1 : 50 | >200 ; confluent |
| | | 1 : 250 | >200 ; confluent |
| | | 1 : 1250 | >200 ; confluent |
| | Virus plus saline | | >200 ; confluent |

Neutralisation : Virus plus serum mixed 1 : 1 and held at 37°C for 1 hour in Experiment 1 and for 4 hours in Experiment 2.

* Earle's saline plus 0·5 per cent lactalbumin hydrolysate.

† Average of 4 replicate plates in Experiment 1 ; and of 3 replicate plates in Experiment 2.

neutralise the haemagglutinating particles. This demonstrates that virus that can produce CPE *in vitro* is neutralised by antibodies against virus that produces tumours *in vivo*. As has already been pointed out in the previous section, virus harvested after CPE produces tumours.

The high early mortality in young virus-injected mice, and the dwarfing effect in these mice after injection, furthermore suggest that a necrotic action of the virus may also occur *in vivo*. It thus seems that both an eventual death and a cell proliferating action can be produced by the same virus, and there is some evidence, which is now being studied quantitatively, that the polyoma can produce cell proliferation *in vitro* before CPE. It could be postulated that after virus infection a cell-virus relationship is established that changes the requirements of the infected cells and that if these new requirements are not met, e.g. as in the tissue culture medium used by us, the cells will degenerate, i.e. a CPE will be produced.

These results with the polyoma therefore provide an experimental analogy to the original observation of Duran-Reynals (1940) on the haemorrhagic disease produced by fowl tumour viruses. A CPE on cells in culture has also been reported for the virus of avian lymphomatosis (Sharpless, Defendi and Cox, 1958 ; Fontes *et al.*, 1958), and the possibility either of growth stimulation or degeneration as a result of infection with the same virus has been observed on the chorioallantoic membrane after infection with the Rous chicken virus (Prince, 1958) and with vaccinia (Overman and Tamm, 1957).

*The production of antibodies in newborn, adult and tumour-bearing animals*

Experiments on immunological tolerance have shown that the antibody-forming system in mice begins to function at about the time of birth (Billingham, Brent and Medawar, 1956), and it was therefore of interest to determine to what extent this is related to the age difference in susceptibility to tumour induction by virus. Using haemagglutination inhibition as a measure of the amount of antibody produced against the polyoma, it has been found that the response to virus infection differs in newborn and adult mice, and that this is reflected in a difference in the induction period before antibodies can be detected (Table V). In adults antibodies could be detected four days after inoculation whereas in newborn inoculated mice in this experiment, the titre was no higher than the control even at eight days. Tests made at later intervals after inoculation have shown that haemagglutination inhibition antibodies have always eventually been produced in mice inoculated as newborn (Table VI) and that there can be high titres even before any palpable tumours are observed. Particularly high titres have however been found in the animals with tumours

These data thus demonstrate that the high susceptibility of newborn mice to tumour induction, does not seem to be due to the production of a state of immunological tolerance to the virus.

It has been shown in a previous section that tumour cells are able to produce virus, and the high titres in the tumour-bearing mice are presumably due to this liberation of virus by cells of the growing tumour. Since the tumours continue to grow despite the presence of high titres of antibody, these data also indicate that antibodies produced against the virus are not effective in preventing the progressive growth of the tumour cells.

TABLE V.—*Induction Period in Newborn and Adult Swiss Mice Inoculated with Virus*

| Days after inoculation | Age of mouse at inoculation | Haemagglutination-inhibition titre 1 : | Number of sera tested* |
|---|---|---|---|
| 1 | Adult | 40 | 3 pooled |
| | Newborn | NT | |
| 2 | Adult | 20 | 3 pooled |
| | Newborn | NT | |
| 3 | Adult | 10 | 3 pooled |
| | Newborn | NT | |
| 4 | Adult | 80 | 3 pooled |
| | Newborn | 40 | 20 pooled |
| 5 | Adult | 320 | 3 pooled |
| | Newborn | 20 | 18 pooled |
| 7 | Adult | 320 | 3 pooled |
| | Newborn | 20 | 6 pooled |
| 8 | Adult | 2560 | 3 pooled |
| | Newborn | 40 | 8 pooled |

All mice inoculated subcutaneously with 0·05 c.c. of polyoma virus with a haemagglutination titre 1 : 256.

NT = not tested.

* Different mice tested each day.

TABLE VI.—*Haemagglutination Inhibition Titres at Various Intervals after Inoculation of Virus into Newborn Swiss Mice*

| Time (in weeks) after inoculation | Number of mice tested | Titre 1 : | Palpable tumours |
|---|---|---|---|
| 2 | 2 pooled | 320 | — |
| 2 | 7 pooled | 1,280 | — |
| 3 | 2 pooled | 640 | — |
| 3 | 2 pooled | 2,560 | — |
| 4 | 2 | 2,560 | — |
| 4 | 1 | 10,240 | — |
| 5 | 2 | 5,120 | — |
| 13 | 2 | >20,480 | + |
| 13 | 1 | 40,960 | + |
| 14 | 1 | 20,480 | + |
| 20 | 2 | >5,120 | + |
| 22 | 1 | >10,240 | + |
| 22 | 1 | >20,480 | + |
| 22 | 1 | 40,960 | + |
| 22 | 1 | >40,960 | + |

\+ = present.
— = absent.

Although the age difference in susceptibility thus does not seem to be due to immunological tolerance, it may still be due to the ability of the virus to multiply and re-infect other cells when inoculated into newborn, to a much greater extent than when inoculated into adults, and this point is being more fully investigated. A comparison of mice inoculated shortly after birth with virus, adult kidney cells followed by virus, and adult spleen cells followed by virus, has shown that at six weeks after virus infection, animals inoculated with the adult antibody-forming cells (spleen) and virus have a significantly greater percentage of survival and marked lack of dwarfing, in comparison to mice inoculated only with virus or with adult kidney cells (i.e. non-antibody-forming cells) and virus.

*Antibodies in animals that develop spontaneous tumours*

The finding of high antibody titres before palpable tumours can be observed, and even high titres in mice with obvious tumours, suggested the possibility that such a situation may also exist in animals which have not been purposely inoculated with virus, but which are known to develop spontaneous tumours. Haemagglutination inhibition tests with polyoma virus have therefore been made on mice of different ages belonging to the high leukaemic strain AKR, and the results are given in Table VII. None of 16 mice less than 23 weeks old had antibody levels higher than the control value, whereas 8 out of 12 mice more than 32 weeks old had considerable antibody titres against this virus. Tests on 20 Swiss mice, 34 to 56 weeks old and 20 C57BL mice 56 to 60 weeks old, have all shown no haemagglutination inhibition in excess of the control value found in younger animals.

TABLE VII.—*Haemagglutination Inhibition Tests with AKR Mice*

| Age in weeks | Number of tests* | Titre 1 : |
|---|---|---|
| 13 | 6 | 20 |
| 15 | 1 | 10 |
| 15 | 5 | 20 |
| 17 | 2 | 20 |
| 23 | 2 | 20 |
| 32 | 1 | 320 |
| 32 | 1 | 640 |
| 32 | 4 | 1280 |
| 34 | 1 | 20 |
| 34 | 1 | 1280 |
| 35 | 2 | 20 |
| 36 | 1 | 10 |
| 36 | 1 | 2560 |

* Each test represents the result from a different mouse.

These data show that AKR mice can carry the polyoma, and furthermore that the detection of antibodies may be a useful guide (*a*) to the existence of tumour virus in animals, and (*b*) to the eventual development of tumours. The absence of high titres in four of the other older AKR suggests either that these animals will not develop tumours, or that although a high antibody titre may frequently be correlated with eventual tumour development, this correlation may perhaps not exist in every animal. The existence of antibodies is therefore being further studied in relation to histological examination of the tissues for malignancy in AKR, and in animals that develop other types of tumours.

In addition to the experiments on AKR, haemagglutination inhibition tests were made with serum from seven C3H mice with large and five with small spontaneous mammary tumours. Of these four with large tumours showed antibody titres of 1 : 80, 320, 1280, and 2560 respectively. Some C3H mice may therefore also carry the polyoma, and since the C3H used was known to carry the mammary tumour virus (milk agent), there may perhaps be some relationship between the mammary virus and polyoma.

*The transmission of polyoma virus*

One of the basic questions relating to tumour viruses is how the viruses are transmitted. In order to obtain information on this point, experiments were carried out with the polyoma on the possibility of virus transmission. (*1*) between litter mates, (*2*) from virus injected litters to their mother, and (*3*) from a virus injected mother to her litter. Haemagglutination inhibition was used as a test for the presence of virus. The results are given in Table VIII, and the data clearly show that virus can be transmitted under all three conditions. It should be noted that in all these tests both the injected and non-injected animals were kept in the same cage. In the experiment in which non-injected animals were kept in the same room but in a different cage (Table VIII) there has so far been no evidence of virus transmission. We have also so far found no haemagglutination inhibition antibodies in the sera of the people who have been in contact with the polyoma in the tissue culture, haemagglutination, and animal experiments.

TABLE VIII.—*Virus Transmission in Swiss Mice*

| Serum from : | Number of mice | Source of infection | Tested (days) after contact | Haemagglutination inhibition titre 1 : |
|---|---|---|---|---|
| Non-inoculated litter mates | 3 pooled | Newborn litter mates inoculated i.p.* | 45 | 1280 |
| Non-inoculated litter | 5 pooled | Mother inoculated i.p. 16th day of pregnancy | 27 | 320 |
| Mother | 1 | Litter inoculated s.c. | 15 | 20 |
| ,, | 1 | Litter inoculated s.c. and i.p. | 15 | 20 |
| ,, | 1 | Litter inoculated s.c. and i.p. | 15 | 20 |
| ,, | 1 | Litter inoculated s.c. | 21 | 80 |
| ,, | 1 | Litter inoculated i.p. | 37 | 80 |
| ,, | 1 | Litter inoculated i.p. | 50 | 320 |
| ,, | 1 | Litter inoculated s.c. | 50 | 640 |
| ,, | 1 | Litter inoculated i.p. | 50 | 640 |
| ,, | 1 | Litter inoculated i.c. | 50 | 640 |
| Adult | 3 pooled | Inoculated with normal tissue culture medium as newborn and kept in same room as virus-inoculated mice | 59 | 40 |
| Control | 5 | None | — | 40 |

* i.p. = Intraperitoneal, s.c. = subcutaneous, and i.c. = intracranial.

It can thus be seen that a tumour virus can be transmitted under certain conditions from animal to animal, and the exact route of infection, i.e. whether from urine, faeces, saliva, milk, transplacental, etc., can now be tested.*

*The isolation of virus from human tumours*

The results described above suggested that by using a similar *in vitro* and *in vivo* approach it may be possible to determine the role of viruses in the origin of human tumours. The methods developed for the mouse are therefore being applied to material from patients. In order to avoid contamination, the experiments are being carried out by one of us in a separate tissue culture cubicle, at a time when he is not handling virus isolated from mice. Human material has been inoculated onto monolayers of Swiss mouse embryos using our standard medium. Cultures consisting of bone marrow from two patients (one with

* *Note added in proof.* Isolations from five animals have shown that the polyoma can be excreted in the faeces, urine and saliva.

myeloid and one with stem cell leukaemia) on Swiss embryo cells have given an effect on the cultures, and the *in vivo* tumour-inducing activity of this material is now under examination.

### DISCUSSION

It can be seen from the above results, that by combining an *in vitro* with an *in vivo* approach, experimental systems can be established in order to answer some of the basic problems relating to the role of viruses in the origin of mammalian tumours. In addition, the establishment of these systems is also of value for the actual isolation and characterisation of tumour viruses.

The development of the plaque and haemagglutination techniques has made it possible to begin a quantitative analysis of cell-virus relationships, which is a basic pre-requisite for an understanding, at the cellular level, of the role of viruses in tumour formation. For this study, the growth of polyoma on mouse embryo monolayers has been used as a model. The finding in this system that a cytopathogenic effect *in vitro* and tumour induction *in vivo* are produced by the same virus, shows that infection with tumour virus can produce under different conditions either growth stimulation or eventual cell degeneration.

The use of *in vitro* tests such as haemagglutination inhibition, has shown that these tests can readily provide information on the presence of tumour virus in an animal, the development of tumours induced by virus, and the transmission of virus from one animal to another.

One of the questions raised in the present studies is the relationship between leukaemia, polyoma, and mammary tumour virus (milk agent), and whether a single or a group of viruses is involved in the origin of multiple tumours. It has been shown that there is a strong similarity between the virus obtained from Gross and polyoma. In addition, the findings (*1*) that high antibody titres against polyoma can exist in some C3H mice (known to carry the mammary tumour virus) with spontaneous mammary tumours, and (*2*) that a high frequency of mammary carcinomas is induced in Swiss mice inoculated with the polyoma, suggest that some C3H mice may carry the polyoma and that this virus may perhaps be related to the mammary tumour virus. The observation of Gross (1955) that extracts from normal C3H tissues can induce parotid tumours and subcutaneous sarcomas when inoculated into newborn C3H mice, also suggests that C3H may carry the polyoma. The fact that the mammary tumour virus cultured on plasma clots with chick fibroblasts (Pikovski, 1953) produced under these conditions only mammary tumours in C57BL mice and C57BL and RIII hybrids, does not necessarily rule out a relationship between this virus and the polyoma. The conditions of the culture system, and possibly even the test animals, may not have been the best to demonstrate the full potentialities of the virus after passage in culture. Mammary tumour virus is therefore being cultured using the same conditions as for the polyoma.

Since with the development of the plaque technique it is now possible to isolate pure lines of virus from different tumours, such isolations are being tested for the types of tumour that they produce in animals. The use of such pure lines in *in vivo* infection experiments should be able to demonstrate conclusively to what extent mammary tumours, leukaemia, and other types of tumours are produced by the same mutants, or quite different strains of virus. The establishment of such pure lines could also provide material for a genetic analysis of these animal viruses. The plaque isolates will thus be able to show to what extent the situation in mice resembles that in chickens where various types of tumours seem to be produced by a group of related viruses (Beard, 1957).

It has been shown that tumour cells can produce virus and this has been demonstrated by placing the cells in culture. This technique may have the additional advantage of providing the most satisfactory method of isolating virus from different tumours and it is now being applied to the studies on humans. In addition, these results suggest that there exists in these tumours some stable form of cell-virus relationship. Evidence for such a relationship has also been obtained for the Rous chicken virus (Rubin, 1955), and it also seems to exist in avian myeloblastosis (Beaudreau *et al.*, 1958). This raises the further possibility that mammalian tumour viruses may prove to be of value for the study of transduction of genetic characters, e.g. antigens, in mammalian cells where there already exist some good genetic markers. There may also exist some further analogues to the fibroma-myxoma transformation (Kilham, 1958). The study of mammalian tumour viruses may therefore be of value not only for an understanding of the origin of tumours, but also for an understanding of some other basic properties of viruses and cells.

### SUMMARY

1. A combined *in vitro* and *in vivo* analysis has beeen made of virus (virus from Gross and polyoma virus) originally isolated from mice with spontaneous lymphatic leukaemia. An *in vitro* study has also been made of a virus from a chloroleukaemia in mice. The *in vitro* analysis has been based on the use of tissue culture monolayers and the development of a plaque and haemagglutination technique. These techniques make possible a quantitative *in vitro* analysis of tumour virus relationships, and the development of plaques further makes possible the production of pure lines of virus. *In vitro* methods can also be applied to the study of human tumours.

2. Using the growth of polyoma on mouse embryo monolayers as a model system, it has been shown that the virus multiplies in tissue culture, that virus is released over a long period of time, that a significant amount of virus produced remains cell associated, and that the data obtained by plaques follows that obtained by haemagglutination.

3. Studies on virus from Gross have demonstrated that there is a strong similarity between a virus from this source and the polyoma, and that at least as regards this tumour, brain is not a better source of virus than leukaemic organs.

4. Virus from chloroleukaemia seems to behave differently in culture than that isolated from mice with spontaneous lymphatic leukaemia.

5. The polyoma when inoculated into newborn Swiss mice produced a range of tumours, the most frequent so far being kidney sarcomas, mucous gland tumours, mammary carcinomas, and hair follicle tumours. The earliest palpable tumours usually appeared about 2 months after inoculation, and in an observation period of 5 months, tumours developed in 90 per cent (61/68) of the inoculated mice. Tumours with a longer latent period were produced in two Swiss mice inoculated at seven days of age, but none in mice inoculated at fourteen days or later. Kidney sarcomas were found two months after inoculation of virus into a newborn rat.

6. Studies on tumour cells in culture have shown that malignant cells can produce virus.

7. It has been shown that a cytopathogenic effect on cells *in vitro*, and tumour induction *in vivo*, are produced by the same virus.

8. Studies on the immune response of the host have shown that the high susceptibility of newborn animals does not seem to be due to the induction of a state of immunological tolerance to the virus. All animals inoculated as newborn eventually develop haemagglutination inhibition antibodies (even before observable tumours) and such antibody titres are particularly high in animals with palpable tumours.

9. AKR mice of different ages (which were not inoculated with virus) were tested for haemagglutination inhibition antibodies against polyoma. None of 16 mice less than 23 weeks old had significant levels of antibody, whereas 8 out of 12 mice more than 32 weeks old had considerable antibody titres. This has shown that AKR mice carry the polyoma, and that the detection of antibodies may be a useful guide to the existence of tumour viruses, and possibly also to the eventual development of tumours.

10. No significant haemagglutination inhibition titres were found in 20 normal Swiss mice 34 to 56 weeks old or in 20 C57BL mice 56 to 60 weeks old.

11. Studies on the transmission of the polyoma have shown that the virus can be transmitted from inoculated to non-inoculated litter mates, from an inoculated mother to her non-inoculated litter, and from an inoculated litter to their non-inoculated mother.

We are very much indebted to the Winfield Baird Foundation for a grant in support of this work.

### REFERENCES

BEARD, J. W.—(1957) *Ann. N.Y. Acad. Sci.*, **68**, 473.

BEAUDREAU, G. S., BECKER, C., SHARP, D. G., PAINTER, J. C. AND BEARD, J. W.—(1958) *J. nat Cancer Inst.*, **20**, 351.

BILLINGHAM, R. E., BRENT, L. AND MEDAWAR, P. B.—(1956) *Phil. Trans.*, s.B. **239**, 357.

DULANEY, A. D., MAXEY, M., SCHILLIG, M. G. AND GOSS, M. F.—(1957) *Cancer Res.*, **17**, 809.

DULBECCO, R. AND VOGT, M.—(1954) *J. exp. Med.*, **99**, 167.

DURAN-REYNALS, F.—(1940) *Yale J. Biol. Med.*, **13**, 77.

EDDY, B. E., STEWART, S. E., YOUNG, R. AND MIDER, G. B.—(1958*a*) *J. nat. Cancer Inst.*, **20**, 747.

*Iidem* AND BERKELEY, W.—(1958*b*) *Proc. Soc. exp. Biol. N.Y.*, **98**, 848.

*Idem*, ROWE, W. P., HARTLEY, J. W., STEWART, S. E. AND HUEBNER, R. J.—(1958*c*) *Virology*, **6**, 290.

ELLERMAN, V. AND BANG, O.—(1908) *Zbl. Bakt.*, **46**, 595.

FONTES, A. K., BURMESTER, B. R., WALTER, W. G. AND ISELER, P. E.—(1958) *Proc. Soc. exp. Biol. N.Y.*, **97**, 854.

FRIEND, C.—(1957) *J. exp. Med.*, **105**, 307.

FURTH, J., BUFFETT, R. A., BANASIEWICZ-RODRIGUEZ, M. AND UPTON, A. C.—(1956) *Proc. Soc. exp. Biol. N.Y.*, **93**, 165.

GRAFFI, A., BIELKA, H. AND FEY, F.—(1956) *Acta Haemat.*, **15**, 145.

GROSS, L.—(1951) *Proc. Soc. exp. Biol. N.Y.*, **76**, 27.—(1953) *Ibid.*, **83**, 414.—(1955) *Ibid.*, **88**, 362.—(1957) *Ibid.*, **94**, 767.

HAYS, E. F. AND BECK, W. S.—(1958) *Cancer Res.*, **18**, 676.

KILHAM, L.—(1958) *J. nat. Cancer Inst.*, **20**, 729.

OBERLING, C. AND GUERIN, M.—(1954) *Advanc. Cancer Res.*, **2**, 353.

OVERMAN, J. R. AND TAMM, I.—(1957) *Virology*, **3**, 173.

PIKOVSKI, M. A.—(1953) *J. nat. Cancer Inst.*, **13**, 1275.
PLOWRIGHT, W. AND FERRIS, R. D.—(1958) *Brit. J. exp. Path.*, **39**, 424.
PRINCE, A. M.—(1958) *Virology*, **5**, 435.
ROUS, P.—(1911) *J. exp. Med.*, **13**, 397.
RUBIN, H.—(1955) *Virology*, **1**, 445.
SACHS, L., FOGEL, M. AND WINOCOUR, E.—(1959) *Nature, Lond.*, in press.
SHARPLESS, G. R., DEFENDI, V. AND COX, H. R.—(1958) *Proc. Soc. exp. Biol. N.Y.*, **97**, 755.
SCHOOLMAN, H. M., SPURRIER, W., SCHWARTZ, S. O. AND SZANTO, P. B.—(1957) *Blood*, **12**, 694.
STEWART, S. E.—(1955) *J. nat. Cancer Inst.*, **15**, 1391.
*Idem*, EDDY, B. E., GOCHENOUR, A. M., BORGESE, N. G. AND GRUBBS, G. E.—(1957) *Virology*, **3**, 380.
*Iidem* AND BORGESE, N. G.—(1958) *J. nat. Cancer Inst.*, **20**, 1223.
WOOLLEY, G. W. AND SMALL, M. C.—(1956) *Cancer*, **9**, 1102.